# Protocol for a Nested Randomized Controlled Trial to Evaluate the Feasibility and Preliminary Efficacy of the Mindfulness Based Health Promotion Program on the Quality of Life of Older Adults Assisted in Primary Care—“The MBHP-Elderly Study”

**DOI:** 10.3389/fmed.2020.563099

**Published:** 2020-11-30

**Authors:** Marcelo Vasconcelos Mapurunga, Solange Andreoni, Daniela Rodrigues de Oliveira, Vicente Sarubbi, Ana Cláudia Bonilha, Vania D'Almeida, Luciana Tomita, Luiz Roberto Ramos, Marcelo Demarzo

**Affiliations:** ^1^Mente Aberta - Brazilian Center for Mindfulness and Health Promotion, Universidade Federal de São Paulo - UNIFESP, São Paulo, Brazil; ^2^Preventive Medicine, Universidade Federal de São Paulo - UNIFESP, São Paulo, Brazil; ^3^Department of Medicine, Universidade Estadual de Mato Grosso do Sul, Campo Grande, Brazil; ^4^Preventive Medicine Department, Centro de Estudos do Envelhecimento (CEE), Universidade Federal de São Paulo - UNIFESP, São Paulo, Brazil; ^5^Department of Psychobiology, Universidade Federal de São Paulo - UNIFESP, São Paulo, Brazil; ^6^Department of Preventive Medicine, Universidade Federal de São Paulo - UNIFESP, São Paulo, Brazil; ^7^Preventive Medicine, Brazilian Center of Attention and Health Promotion, Universidade Federal de São Paulo - UNIFESP, São Paulo, Brazil

**Keywords:** quality of life, elderly, MBHP, mindfulness (MeSH, NHL), health promotion (source: MeSH NLM), primary care (MeSH)

## Abstract

**Introduction:** Population aging is a global phenomenon that has grown rapidly and progressively all over the world. Interventions that promote health must be studied and implemented to make the aging process be with quality of life. Depression and anxiety are the most common mental health conditions that compromise the quality of life on the elderly and it can cause damage to the autonomy and activities of daily life. Mindfulness training has been shown to improve psychological health and quality of life on adults. Studies involving Mindfulness-Based Interventions (MBIs) with older people are scarce in the literature, but they have been increasing in recent years showing promising results for healthy aging. This trial will investigate the feasibility and preliminary efficacy of an MBI on the quality of life of elderly assisted in the Primary Care.

**Materials and Methods:** A cohort-nested randomized controlled trial with 3 assessment points (baseline, post-intervention and 1-year follow up) will be conducted to compare a MBI program (Mindfulness-Based Health Promotion) to a cognitive stimulation control-group in a Primary Care facility. One-hundred and two older adults will be recruited from a cohort of this facility and they will be randomized and allocated into an intervention group (*N* = 76) and the control group (*N* = 76). The primary outcome evaluated will be the improvement of quality of life assessed by the WHOQOL-BREF and WHOQOL-OLD. The secondary outcomes will be cognitive function, psychological health, sleep quality, self-compassion, and religiosity. Qualitative data will be assessed by focus group and the word free evocation technique. The feasibility of the program will also be evaluated by adherence and unwanted effects questionnaires.

**Discussion:** This cohort-nested clinical trial will be the first mixed-methods study with 3 assessment points which will study the feasibility and preliminary efficacy of a mindfulness-based program for older people in Latin America population. If the findings of this study confirm the effectiveness of this program in this population it will be possible to consider it as intervention that might be implemented as public policy addressed to older people in healthcare systems.

**Clinical Trial Registration:** www.ClinicalTrials.gov, identifier: NCT03048708. Registered retrospectively on October 11th 2018.

## Introduction

Population aging is a global phenomenon that has grown rapidly and progressively all over the world because of factors such as increased life expectancy and declining birth rates. Brazil has followed this trend with a speed slightly higher than the world growth pattern (1), which has called attention both in scientific research and in the perspective of necessary public policies that promote health and quality of life to attend this demand.

The concept of quality of life is complex to be defined, and it can be interpreted in a multidisciplinary way due to the different cultural, ethical, religious, and people values that influence the way it is perceived (2). The definition of quality of life, according to the World Health Organization Quality of Life Group, is related to “individuals' perception of their position in life in the context of the culture and value systems in which they live and in relation to their goals, expectations, standards, and concerns” (3). From this conceptualization, we can understand that the perception of quality of life has its subjective, multidimensional, and contextual character for each individual.

There is no clear consensus regarding the definition of quality of life in the elderly (4). However, García and Navarro say that quality of life for the older people is a multidimensional concept that is related to their life satisfaction, autonomy, and independence in their execution of their activities of daily living (ADLs) (4). In terms of health assessment, tasks that involve their functional capacity, autonomy, and independence are known as ADLs, and it refers to basic activities related to self-care such as feeding, bathing, and dressing, as also instrumental activities lead an independent life within the community like going shopping (5). Studies show that the better the ADLs, the better the quality of life (6–9).

A longitudinal study published (10) demonstrated that older people who meditated more frequently over 59 weeks between the initiation of an 8-week mindfulness-based intervention (MBI) program and the 1-year follow-up had a greater performance in their ADL than the group that meditated less frequently.

The MBIs were originated from the mindfulness-based stress reduction (MBSR) program. The MBSR was built by Jon Kabat-Zinn et al. at the University of Massachusetts Medical Center (11, 12), and it is an intervention whose effects on health promotion and quality of life have produced several studies worldwide (10, 13–21). Recently, its implementation in primary care (PC) (22, 23) and its benefits with the elderly population (10, 24–31) have been studied.

*Mindfulness* can be defined as a state or trait that refers to the ability to be aware of what happens in the present moment intentionally and non-judgmentally, and it involves two fundamental components: self-regulation of attention and open orientation to one's own experience with curiosity, openness, and acceptance. One of the most used ways to get to this state of mindfulness is through the formal practice of mindfulness meditation within MBI programs (11, 32, 33).

A study has demonstrated that an MBI program has reduced the perception of loneliness in the elderly (24). Loneliness is a risk factor associated with decreased cognitive function, depression, and morbid and unhealthy behaviors in the aging population (34, 35). Depressive and anxiety disorders are the mental health conditions that most compromise the quality of life of the elderly (36, 37). According to Krug et al. (38), physical, cognitive, and social activities reduce the consequence of cognitive decline and can be used as preventive measures.

Several protocols have been developed based on the MBSR aimed at specific samples, such as the Mindfulness-Based Health Promotion (MBHP) (39) program developed by the Mente Aberta – Brazilian Center for Mindfulness and Health Promotion. The MBHP program was inspired by the original MBSR model but adapted to the context of PC and health promotion, and it has been applied in Brazil.

Despite the increasing number of researches investigating the effects of MBIs with the elderly (26, 27, 40–42), they are still scarce, and most are non-randomized and inconclusive, with small sample size and with high risk of bias (43). Thus, more controlled, randomized, and mixed-methods studies involving MBIs with the elderly are needed, especially with experiences of its applications in PC, a core component of healthcare systems, both public and private.

Thus, the aim of this study is to evaluate the feasibility and preliminary efficacy of the MBHP program on quality of life (primary outcome) of older adults assisted in PC comparing to a cognitive stimulation active control group. Our main hypothesis is that the MBHP is feasible and efficacious in promoting quality of life in people older than 60 years, and it will improve psychological health, self-compassion level, sleep quality, religiosity, and score at ADLs and prevent or delay cognitive decline in the elderly (secondary outcomes). Also, we hypothesize that the MBHP is superior to a cognitive stimulation group to promote those benefits.

## Materials and Methods

The “MBHP-Elderly Study” is a randomized controlled trial addressing older people with 3 assessment points (baseline, post-intervention, and 1-year follow-up) nested in a cohort study (the EPIDOSO study) of older adults patients assisted in a PC facility of the “Center for Aging Research” [Centro de Estudos do Envelhecimento (CEE)] of the Universidade Federal de São Paulo—UNIFESP.

**Figure 1:**
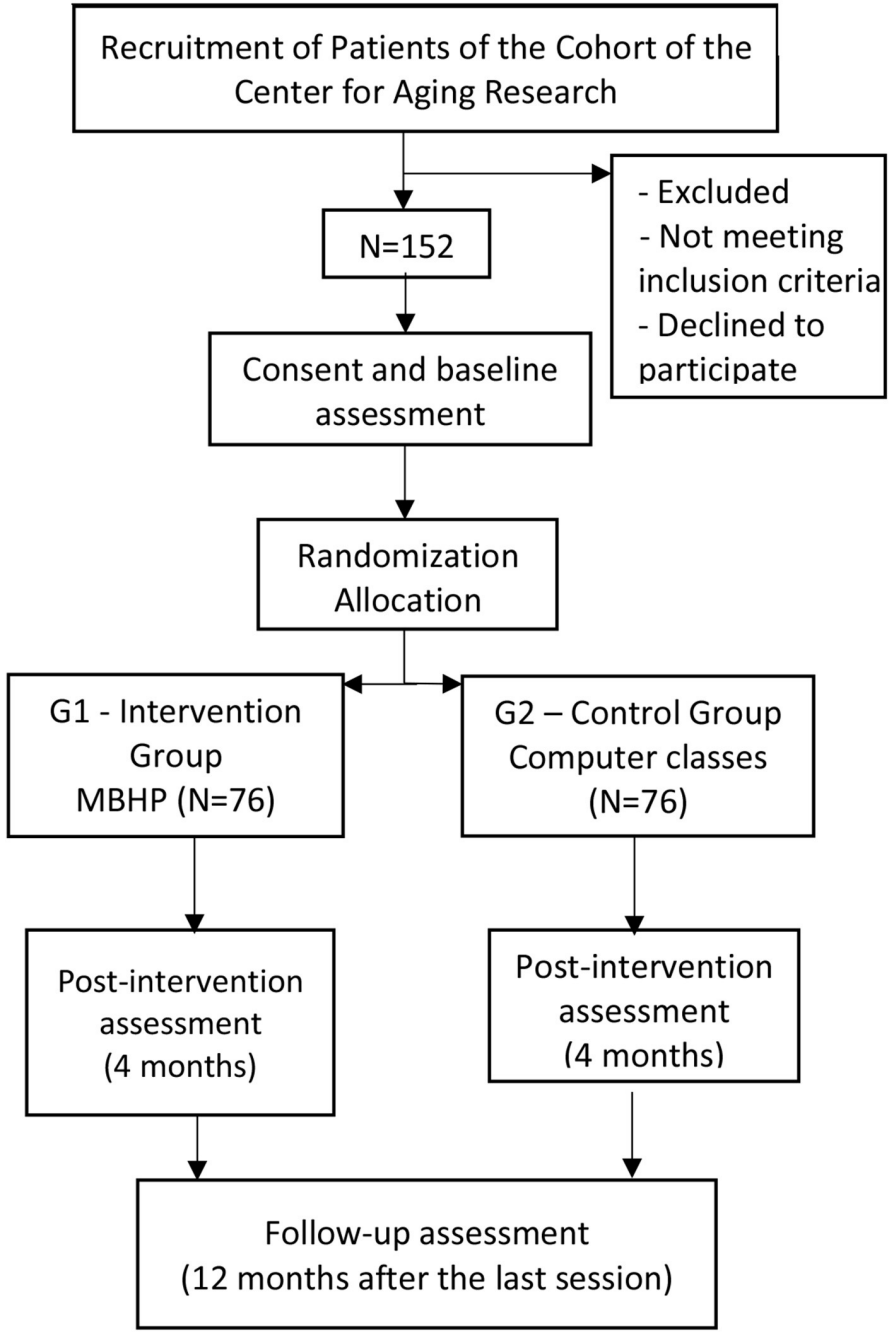
Diagram of planned study flow of participants and study design.

One hundred fifty-two participants will be recruited from the cohort and then randomized into the following two experimental groups: (I) MBHP intervention group (76 participants) in which the elderly will have a weekly meeting of an hour and 30 min for 8 weeks to perform the MBI (MBHP protocol) accompanied by extra four 1-h meetings through a maintenance group with all participants after the eighth week (each maintenance session took place fortnightly), totaling a 4-month (16-week) intervention; (II) cognitive stimulation control group (76 participants) of elderly who will take computer-based cognitive stimulation classes for 4 months, once-a-week 1.5-h meetings without maintenance phase, totaling 16 weeks. In both groups, the same assessment for data collection will be applied, and they will be applied in the baseline (T0), post-intervention (T1), and 1-year follow-up (T2). Both interventions will last 16 weeks. However, the reason why the MBHP intervention group will take 12 sessions and the cognitive stimulation control group 15 sessions is because one of the aims in the MBHP intervention is to work on the participants' autonomy in their personal mindfulness practice in daily life through home activities exercises, and we wanted to compare its outcomes with the 4-month weekly meetings of control intervention, which require face-to-face sessions. Study design and participant flow through the trial is shown in Figure 1.

We have decided to use an active control group only (instead to an additional waiting-list control group) due to ethical reasons and to facilitate the recruitment for the study, based on previous protocols' experience of our research centers.

**Figure 2:**
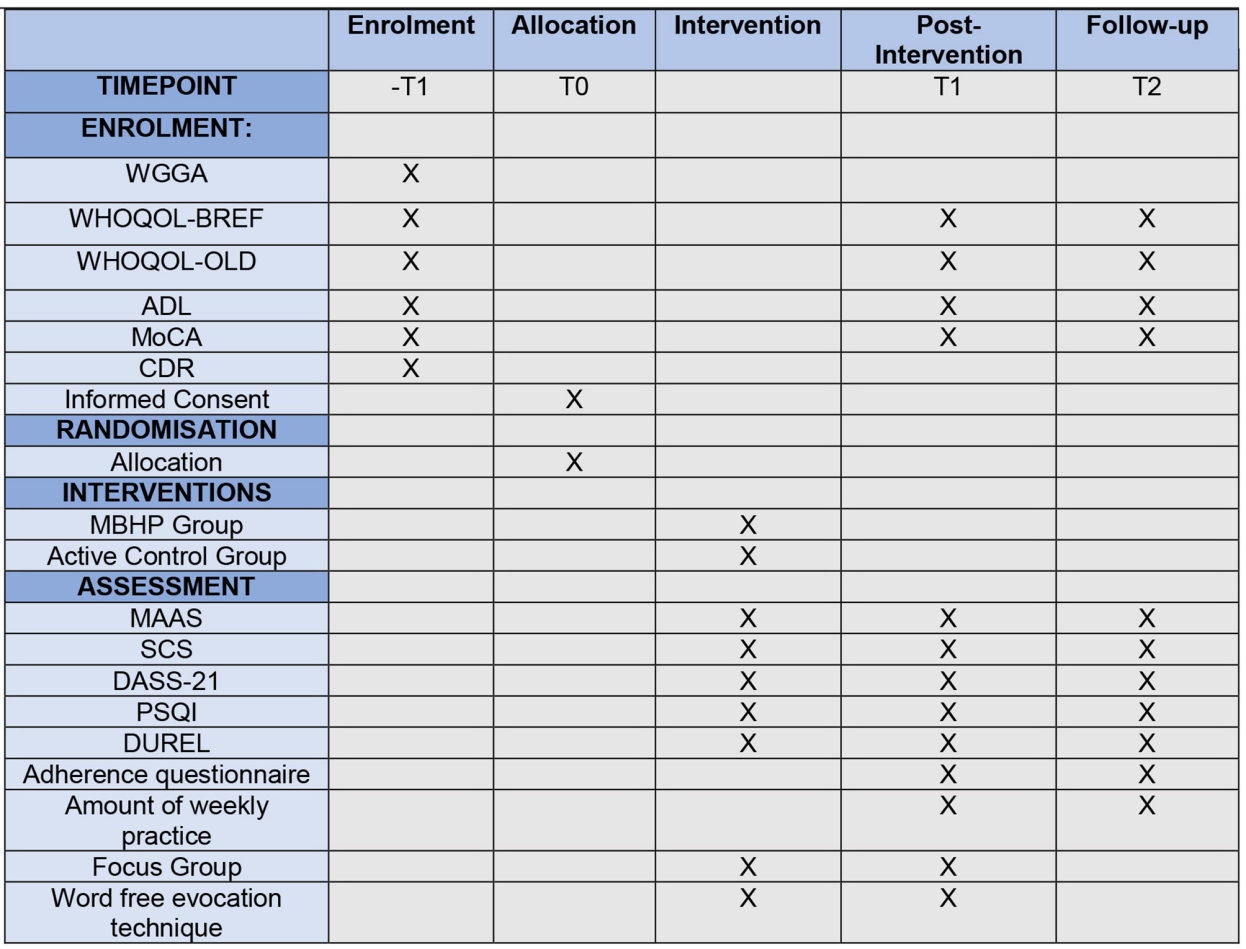
Standard Protocol Items: Recommendations for Interventional Trials (SPIRIT) diagram. ADL, Activities of Daily Living Scale; CDR, Clinical Dementia Ratings; DASS-21, Depression Anxiety Stress Asessment-21; DUREL, Duke Religious Index; MAAS, Mindful Attention Awareness Assessment; MBHP, Mindfulness-Based Health Promotion; Active Control Group, Cognitive Stimulation; MoCA, Montreal Cognitive Assessment; PSQI, Pittsburgh Sleep Quality Index; SCS, Self-Compassion Scale; -T1, Screening and Recruitment; T0, Before Intervention; T1, Post-Intervention; T2, 1-year follow up; WGGA, Wide Geriatric and Gerontologic Assessment; WHOQOL-BREF, World Health Organization Quality of Life–BREF; WHOQOL-OLD, World Health Organization Quality of Life–OLD.

The steps of the study will be conducted based on Consolidated Standards of Reporting Trials (CONSORT) guidelines to report clinical studies in a clear, transparent, and comprehensive manner (44). The protocol is reported according to the Standard Protocol Items: Recommendations for Intervention Trials (SPIRIT) statement [(45); Figure 2], and it was approved by the center ethical committees. This study was retrospectively registered under ClinicalTrials.gov NCT03706807 on October 11, 2018.

### Sample Size and Power Calculations

The minimum sample for each group was identified as 64 participants to detect a power of 0.80 and an α of 0.05. These calculations were performed using the G^*^Power 3.1 software for comparison between two intervention groups with respect to the mean differences of the scores at each time point of assessment relative to baseline. Therefore, 152 individuals should be randomized to receive one of the interventions (76 in the intervention group, 76 in the control group) assuming a maximum loss of 15%.

### Participants and Settings

The samples will comprise 152 participants of the cohort study of the Center for Aging Research (CSR) of the UNIFESP. The CSR is responsible for the EPIDOSO (“Epidemiologia do Idoso”) study, which is a cohort of elderly population living in the community district Vila Clementino in the city of São Paulo, Brazil. The EPIDOSO is the first longitudinal study in Latin America that aimed to identify factors that would influence healthy aging in elderly residents of the community. Vila Clementino is a neighborhood with homogeneous population without great significance of migration into or out of the neighborhood and represented by majority middle class population.

### Cohort Wave

Between December 2007 and April 2008, the CSR draws a random sample of 2,000 people 60 years or older living in a middle-class neighborhood, with high educational level and low migration rates, in the city of São Paulo, for a multicentric household survey to investigate the validity of the ADLs to assess need for help (46). After the home interview, all interviewees were invited to participate in an open cohort. A total of 1,155 elderly attended the invitation at that time and had a comprehensive geriatric and gerontological evaluation at the CSR of the university, which provided data on sociodemographic characteristics, referred chronic diseases, medications in use, cognitive status, and independence in daily life.

### Participants' Enrollment

Between June 2018 and May 2019, we contacted the participants of this cohort by phone calls, and they were invited to participate in the present study. We booked those who agreed for a new application of the comprehensive geriatric and gerontological evaluation, now including the Clinical Dementia Rating (CDR) scale (47, 48) applied by a trained professional through an unstructured interview with a relative of the participant. This enabled the selection of individuals with normal cognition (CDR = 0) or mild cognitive impairment (CDR = 0.5) in the study.

The participants of the cognitive stimulation and mindfulness trial answered a specific protocol that included Montreal Cognitive Assessment (MoCA) and a questionnaire to find out what computing level they had and if they have already experienced other sorts of mediations practices on the last 6 months.

After signing the consent form, we randomly assigned study subjects to two comparative groups: intervention and control, with an allocation ratio 1:1, following the sequence of arrival in the study as shown in Figure 1.

### Eligibility

To be admitted in the study, participants will meet the following inclusion criteria: (a) men and women 60 years or older; (b) elderly classified as cognitively normal (CDR = 0) and/or with mild cognitive impairment (CDR = 0.5); (c) be literate; (d) not have an advanced computer level; and (e) have a good hearing to follow the practices.

Exclusion criteria are as follows: (a) participants performing contemplative practices such as yoga, tai-chi-chuan, vipassana, Zen Buddhism, mindfulness, and other meditative practices in the last 6 months; (b) patients with acute phase of depression evaluated by the Geriatric Depression Scale (GDS-15) (49–51); (c) patients with psychotic diagnosis; (d) who are taking drugs that cause cognitive impairment; (e) participants who score 1.0 or more at the CDR (47, 48, 52) applied during the recruitment by a trained gerontologist in order to diagnose cognitive impairment.

### Instructors

Four mindfulness tutors will lead the MBHP groups—on Mondays, Tuesdays, Wednesdays, and Thursdays, respectively. All of them were already certified by the Mente Aberta – Brazilian Center for Mindfulness and Health Promotion and have at least 2 years of experience leading the MBHP protocol at the time of the study. The Mente Aberta certifying professional training follows the British and Brazilian guidelines for good practices of mindfulness (53). The cognitive stimulation control group will be conducted by an experienced gerontologist of the CSR who had a specific training in the protocol (described in detail below).

### The MBHP Protocol

The intervention in the MBHP-Elderly Study will be the MBHP program [(39); Table 1]. It was developed by Mente Aberta Center. It is originally an 8-session (2 or 1.5 h each) program based on the MBSR model created by Kabat-Zinn et al. (11). The MBHP is adapted to the context of PC with a framework that supports the learning process to individuals from different cultures and education backgrounds (54). It emphasizes exercises to be carried out at home or work environment in order to incorporate mindfulness into their daily lives through formal practices such as meditations and informal practices such as walking, eating, and talking with awareness. It also emphasizes the concepts of radical acceptance, values clarification, and positive psychology (54).

**Table 1:** Mindfulness-Based Health Promotion (MBHP) program.

**Session**	**Practices**	**Homework**
1. Leaving the automatic pilot	Raisin exercise—body scan	Body-scan-mindful eating−9 dots exercise
2. Mindfulness in the body	Body scan—primary and secondary suffering—exploring breath—mindful breathing	Body scan-mindfulness in breathing-awareness of pleasant events (diary)—attention for routine activity and habit changing
3. Mindfulness in movement	Mindfulness in breath—hello, thank you, goodbye—mindful walking	Mindful breathing—mindful walking-awareness of unpleasant events (diary)—attention for routine walking
4. Expanding mindfulness	Mindful movements—mindfulness of sounds and thoughts−3 min practice	Sounds and thoughts practices−3 min practices—awareness of communication difficulties (diary)
5. Dealing with difficulties	Sounds and thoughts practice−3 min breathings space in doubles	Sitting meditation—body scan—movement practice—sound and thoughts practice−3 min practice at a challenge situation
6. Mindfulness and silence	Body scan—mindful breathing—mindful walking—mindful movements	Practice of choice—mindful conversation
7. Compassion	Sound and thoughts practice—sitting meditation—loving-kindness (for oneself and other)	Practice of choice—compassion practice
8. Mindfulness for life	Self-compassion practice—connection with values	Daily life mindfulness incorporation

The main techniques used in this program are “mindfulness in breathing,” “body scan,” “mindful walking,” “mindful movements,” and “3 min of mindfulness” (adapted from the program of the Breathworks Institute, UK). It also introduced the concepts of “first and second suffering” and the “Hi-Thanks-Bye” as psychoeducation techniques. The compassion and self-compassion practices are also included in the protocol, which might be useful for older people dealing with the self-criticism and low levels of acceptance of body changes. There is no consensus about potential unwanted effects of MBIs in the literature yet (55).

In this study, the MBHP sessions will be adapted and abbreviated for 1 h and a half per week for 8 weeks. After the 8-week protocol, four 1-h maintenance sessions will be performed every 15 days to match a 4-month intervention time period in full. We chose this format and length to be able to compare with the active control group (as described below). Moreover, studies that applied MBIs for longer months with older adults have been previously reported and may be beneficial to them (56, 57), as well as meeting lasting 1 h and 30 min instead of 2 h during the 8-week program (29, 30).

### Cognitive Stimulation Protocol

The active control group will receive a cognitive stimulation training in the CSR facility. This training is a computer class workshop based on a model proposed by Xavier et al. (58), which uses the staging methodology considering cognitive and functional resources accordingly to the level of complexity and difficulties from the executive functions theory. The basic workshop is considered a cognitive stimulation, and the participants will receive a class 1 h and 30 min once a week for 16 weeks (4 months).

The abilities learned at the workshop will be basic computer functions from development of the psychomotricity involving using mouse, keyboard, MS Paint, photo gallery, and PowerPoint presentations; surfing on the internet; and learning how to play online games and use social networks. The activities of the workshop are elaborated and adapted accordingly to the development of each class by the participants so that they get the same group level.

### Recruitment, Procedures, and Randomization

Overall, 152 elderly were recruited from the cohort of the CSR of the Universidade Federal de São Paulo from June 2018 to May 2019. Recruitment was conducted by invitation phone calls to the cohort list. Participants who showed interest in the research were scheduled to visit the center so that one of five researcher assistants would apply a comprehensive geriatric and gerontological evaluation named as Wide Geriatric and Gerontologic Assessment (WGGA), which comprise sociodemographic data and other measures' information needed for the recruitment such as GDS-15 (49–51) and baseline data such as quality of life [World Health Organization Quality of Life (WHOQOL) BREF and WHOQOL-OLD] (59–62) and ADLs, and then the MoCA (63, 64) is applied in order to evaluate cognitive function.

After this first step, a trained gerontologist applies the CDR (47, 48, 52) to evaluate if the participant has dementia or any cognitive impairment. The CDR evaluation requires a caregiver or a relative close to the older adult to be interviewed to inform about his cognition and behaviors (48). The participants who score 0 or 0.5 were included in the study.

After the screening and recruitment, the 152 participants approved in the inclusion criteria will be randomized and allocated into the groups. Married participants who meet the inclusion criteria will be allocated to the same groups; it was decided to do that because some couples need the support from one another to go to the center. The MBHP program group will then be randomized again into other four subgroups (19 participants each) because the MBI group is better availed when smaller. All the groups will follow the same assessment of the research, and the MBHP protocol will be led by instructors certified by Mente Aberta the whole time. The control group will also be randomized and allocated into small groups according to the number of computers in the computer lab (19 participants each).

Randomization will be implemented using the Microsoft Excel. Each participant will be randomized individually for each of the two groups. The couples will be stratified into the same groups as it was also considered that, if they were allocated to be separated, one could teach another what was taught and then contaminate the sample. All randomization procedures will be made by a statistical professional who will only receive the identification number of the participants and will not interact with them at any time before, during, or after the intervention.

After the randomization, the participants will be invited to attend the CSR for a lecture, which we will inform which group they were randomized to and when the intervention will start. At this moment, we will hand the participants the informed consent in written form to be signed by the interested and the measure scales that were not filled during the screening such as Mindful Attention Awareness Scale (MAAS) (65, 66), Self-Compassion Scale (SCS) (67, 68), Depression Anxiety and Stress Scale (DASS-21) (69, 70), Pittsburg Sleep Quality Index (PSQI) (71, 72), and Duke Religious Index (Durel) (73, 74).

Due to the nature of the intervention, this will be a single-blind study, in which the outcomes assessment and analyses will be blind, but the participants will be aware of their group assignment.

### Assessment and Outcomes

The first two assessment points (baseline, post-intervention) will occur over a week before the intervention and on the last meeting of the maintenance group, and the follow-up will occur 1 year after the last meeting of the intervention. For the baseline, the sociodemographic questionnaire, WHOQOL-BREF, WHOQOL-OLD, MoCA, and ADL questionnaire occurred during the recruitment; all the other scales will be applied a week before the intervention starts. The measures of the intervention group will be on sheets of paper as most of the participants are not familiar with using electronic devices. The typing of the data will be made by two people to supervise and to guarantee no typing mistake of the collected data. SurveyMonkey software will be used for data collection for control group as the participants will learn how to use it on the cognitive stimulation classes. All the instruments have been validated into Portuguese with reliable psychometric properties and are available in Supplementary Files (48, 49, 60, 61, 63, 65, 68, 70, 71, 73).

### Geriatric and Gerontologic Interview

During the recruitment, trained professionals on the field of gerontology, nutrition, psychology, and medicine applied the WGGA developed by the CSR in order to collect sociodemographic data and evaluate the health, ADLs, chronic diseases, the drugs taken, mental health, life satisfaction, and cognitive function of the elderly. WGGA is based on the BOMFAQ (Brazilian OARS Multidimensional Functions Assessment Questionnaire) (75) in order to evaluate ADL, on the MHSQ (Mental Health Screening Questionnaire) (76) in order to evaluate mental health, and on the MMSE (Mini-Mental State Examination) (77) in order to evaluate cognitive function. The WGGA also include GDS-15 (49–51) in order to evaluate depression and WHOQOL-BREF (61) with WHOQOL-OLD (60) in order to measure quality of life. In this study, we will use GDS-15 only for inclusion criteria, in which the participants who score 7 or more will be excluded.

### Primary Outcomes Measure

#### WHOQOL-BREF

Developed by the World Health Organization, the instrument is a short version of WHOQOL-100, and it comprises 26 items in which two are general about quality of life and general health, and the other 24 grouped into four domains: physical health, psychological health, social relationships, and environment. It presents Likert scale answer (1–5 points). The Brazilian validation of WHOQOL-BREF Cronbach coefficient values has satisfactory internal consistency ranging from 0.69 to 0.84 between the domains (61).

The WHOQOL-OLD module will be used as a complement to the WHOQOL-BREF to evaluate the quality of life of the elderly. The WHOQOL-OLD follows the same methodology as WHOQOL-100, but the objective is evaluating the specific quality of life of the elderly population. The module comprises 24 items evaluated by the Likert scale (1–5 points) assigned six facets such as sensory abilities, autonomy, past–present–future activities, social participation, death and dying, and intimacy. The Brazilian validation of WHOQOL-OLD also has a satisfactory internal consistency with its Cronbach coefficient between 0.71 and 0.88 (60).

### Secondary Outcomes Measures

#### MAAS

It is a unidimensional 15-item scale that assesses the level of mindfulness or consciousness in the present moment of the individual (66). It was validated in Brazil by Barros et al. (65), and it has a satisfactory internal consistency and reliability with the Cronbach coefficient of 0.83.

#### SCS

It was developed by Neff (67) to measure the level of self-compassion of the individual. The SCS consists of 26 items divided into six subscales for self-kindness, self-judgment, mindfulness, common humanity, and isolation. The answers are given in 5 points of the Likert scale, which (1) is almost never and (5) is almost always. The Brazilian validation was made by Souza and Hutz (78), and it has good internal consistency with Cronbach α of 0.92 for the 26 items.

#### DASS-21

In order to assess psychological health, we will use the DASS-21. It is a 21-item scale that measure and divide the symptoms of anxiety, depression, and stress, in which participants indicate the degree to which they experienced these symptoms in the previous week on 7 items in each subscale. It is a Likert scale of 4 points between 0 and 3. The highest score in each subscale refers to more negative affective states (69). It was adapted into the Brazilian Portuguese language by Vignola and Tucci (70) presenting Cronbach α of 0.92 for the subscale of depression, 0.90 for the stress, and 0.86 for anxiety. Gloster et al. (79) suggest that DASS-21 is an effective instrument when applied in the elderly population.

#### PSQI

The PSQI is an instrument that evaluates the sleep quality of individuals in the last month. It comprises 19 questions in self-report and 5 questions answered by some third party in which it will be made by phone call to a caregiver, relative, or spouse (72). It was validated for Brazilian population (71), and it showed a good internal consistency with Cronbach α of 0.82. The components of the PSQI are subjective sleep quality, sleep latency, sleep duration, habitual sleep efficiency, sleep disturbance, use of sleeping medication, and daytime dysfunction. Categorized as seven components in scores from zero (no difficulty) to three (severe difficulty). The sum of the results can range from 0 to 21 scores, where the higher the number, the worse the quality of sleep. A total score of <5 indicates that the individual is experiencing difficulty in at least two components or moderate dysfunctions in at least three (80).

The Duke University Religion Index is a 5-item scale that measures three major dimensions of religiousness: (1) organizational, the frequency of attending public religious activities and group-related; (2) non-organizational, which involves religious activities performed in private, such as prayer, scripture study, watching religious TV, or listening to religious radio; (3) intrinsic religiosity, assessing the degree of personal religious commitment or motivations (74, 81), and it has adequate internal consistency in the Brazilian version (73).

#### MoCA

This neuropsychological test assesses the cognitive function. It is a 12-item test that measures eight cognitive domains: short-term memory (delayed drawing), visuospatial abilities (cube drawing, clock drawing), executive function (trail-making test, phonemic verbal fluency, verbal abstraction), attention, concentration, working memory, language, and orientation to time. The test total score is 30 points where a score ≤26 indicates normality, and less indicates mild cognitive impairment (63, 64).

At the post-intervention, the feasibility and adherence of MBHP program among the participants will be assessed through a questionnaire created by our research group in order to evaluate the type, frequency, and length of personal meditative practice at home during the week out of the meetings, addressing potential unwanted effects as well. The frequency of the attendance to the weekly meeting will also be monitored. The dropout cases will be monitored, and we will make phone calls to them to ask the reasons why they were absent or did not go on attending the meetings. For more information about the target of each scale assessment and its time points, see Table 2 and Figure 2 to see each step of the study.

**Table 2:** Overview of measures and time points (according to 2013 SPIRIT figure guidelines).

**Measure**	**Target**	**T0**	**T1**	**T2**
		**Baseline**	**Post-intervention**	**Follow up**
WHOQOL-BREF	Quality of life	X	X	X
WHOQOL-OLD	Quality of life of elderly	X	X	X
MoCA	Cognitive function	X	X	X
CDR	Dementia	X		
ADL	Activities of daily living	X	X	X
MAAS	Mindfulness trait	X	X	X
SCS	Self-compassion	X	X	X
PSQI	Sleep quality	X	X	X
DUREL	Religiosity	X	X	X
DASS-21	Psychological health	X	X	X

*ADL, Activities of Daily Living Scale; CDR, Clinical Dementia Ratings; DASS-21, Depression Anxiety Stress Asessment-21; DUREL, Duke Religious Index; MAAS, Mindful Attention Awareness Assessment; MoCA, Montreal Cognitive Assessment; PSQI, Pittsburgh Sleep Quality Index; SCS, Self-Compassion Scale; WHOQOL-BREF, World Health Organization Quality of Life–BREF; WHOQOL-OLD, World Health Organization Quality of Life–OLD*.

### Statistical Analysis

All analyses will be conducted using intention-to-treat and per-protocol analyses following the CONSORT recommendations for reporting the results (44).

Categorical variables will be described by absolute and relative frequencies. Numerical variables will be checked for their distributions through histograms and boxplots. If considered as having normal distribution, they will be described by mean and standard deviation. Otherwise, they will be described by medians and quartiles.

The primary outcome of this study is the quality of life measured by the WHOQOL-BREF and WHOQOL-OLD, and the secondary outcomes are cognitive function measured by the MoCA, level of consciousness at present measured by the MAAS, self-compassion measured by the SCS, Psychological Health measure by DASS-21, sleep quality measured by PSQI, and religiosity assessed by the Duke University Religion Index. Sociodemographic questions (sex, age, schooling), treatment group, and time will be the independent variables.

To verify the homogeneity between the study groups regarding sociodemographic measures at the initial time of the study, the groups will be compared by means of hypothesis tests of the type: χ^2^ and/or Fisher exact for categorical variables and multivariate analysis of variance (MANOVA) and/or Kruskal–Wallis for numerical or ordinal variables. A descriptive analysis of the sociodemographic categories regarding the sex, age, and schooling of both groups will be carried out.

Measures of preintervention (T0), post-intervention (T1), and follow-up (T2) outcomes may be compared by means of MANOVA for repeated measures. If necessary, Student *t*-tests will be conducted for paired or Wilcoxon data by applying corrections by the Holm or Bonferroni method. A Pearson correlation coefficient analysis will also be performed between the means of the primary outcomes and those of the secondary outcomes to examine the degree of changes in the outcome measures over time.

The difference between the measures T0 to T1, T1 to T2, and T0 to T2 can be compared between the intervention groups in MANOVA of repeated measures by means of Mann-Whitney tests. If necessary, these models will also be adjusted to assess the effects of time and intervention group, controlling for possible moderating variables.

### Qualitative Assessment

Focus groups with a minimum of 4 and a maximum of 10 participants will be performed a week after the last session; semistructured questions will be asked in order to evaluate their quality of life perception before and after the interventions. The participants invited into the focus group will be randomly chosen from a list of those who attended at least 75% of the sessions.

All the focus groups will be led by a collaborator of the research with experience conducting these sorts of groups. The content of the speeches will be fully recorded in digital audio, and then all the data will be transcribed. We expect this analysis to identify the feasibility and adherence and non-adherence to mindfulness and its health effects.

We will also apply the free evocation of words technique during the qualitative assessment. It consists of asking the interviewer to say five words (simple or compound) that come to mind when listening to the inducing terms: *body*, *aging*, *quality of life*, and *meditation*, and then it is asked to mark which of the words evoked is the most important and why. This assessment will be done on paper with the lines for the participants to write the five words and the explanation. Evocation will be organized in order of frequency and appearance. This technique is used to characterize social representation in which the frequency and orders of word evocation are calculated (82).

### Qualitative Analyses

The word evocations will be organized in order and frequency of appearance with the help of the EVOC 2005 software (83). They will be analyzed by their frequency and the order of the evoked sequence, estimating their evocation mean order. The order and frequency in which the first words evoked were evoked demonstrate the most strongly associations linked to collective affections and imaginary.

The justifications content of the words obtained in the evocations, as well as in the focus groups, will be analyzed from the thematic–categorical content analysis technique, considering the analytical categories and apprehension of the empirical categories arising from the content of the speeches. The technique of content analysis aims to describe the manifest content objectively, systematically, and quantitatively with the purpose of its interpretation (84). NVivo software version 12 will be used to aid in the processing, organization, coding, and thematic analysis.

### Ethical Considerations

This study was registered under ClinicalTrials.gov as NCT03706807 retrospectively on October 11, 2018, and it was submitted to the Research Ethics Committee/Plataforma Brasil–UNIFESP and approved under CAAE 89700318.0.0000.5505 and opinion number 3.557.601. The SPIRIT statement recommendations for clinical trials were adopted (45). The participants of the research will receive a written informed consent to be signed.

### Trial Status

At the time of this manuscript submission, the recruitment for the MBHP-Elderly Study was already ongoing (started in June 2018 and predicted to finish in February 2021 with the follow-up collection).

## Discussion

By the progressive increase of the elderly population in Brazil and all over the world, it is necessary to study accessible, feasible, and efficacious interventions that promote healthy aging and improve quality of life of this population. Research on MBIs with older participants is still scarce in the literature. The MBHP-Elderly Study will assess the effects of the MBHP program on quality of life (primary outcome), cognitive function, psychological health, mindfulness, sleep quality, self-compassion, and spirituality at three assessments points (preintervention, post-intervention, and follow-up) to investigate the potential gains of the intervention and if it is maintained over time. To the best of our knowledge, the MBHP-Elderly Study is the first to propose such research question in a Latin population considering its own cultural and healthcare aspects.

Mindfulness-based interventions have been associated with quality of life in adults in several studies (14, 16, 85, 86), and a previous study has shown high levels of adherence to an MBI in Brazil (13). According to the literature, people who practice mindfulness in their lives have fewer symptoms of anxiety and depression and deal better with dysfunctional stress and chronic pain (13, 20, 21). Therefore, we believe that these interventions can have positive outcomes in improving the quality of life of the elderly assisted by the PC, and in this study, we will also monitor the level of adherence of the older adults at post-intervention and 1-year follow-up.

We hope to demonstrate, assuming our priori hypothesis is true, that health promotion effects of MBHP are also valid for the elderly population. There are a few studies that show the effect of MBIs on older adults, but controlled and randomized clinical trials are scarce (43), and especially with mixed methods. Moreover, there is not any study that evaluated MBHP protocol directed at older people yet. This will be the first study of the MBHP with older people with qualitative and quantitative method assessments. If the findings of this study confirm the effectiveness of this program in this population, it will be possible to consider it as intervention to be implemented in other health facilities and as a public policy addressed to older people.

One potential limitation of the study is the lack of a second and passive control group composed of a waiting-list participant, besides the active control group. There is a growing discussion over the use of waiting-list control group in psychological and behavioral research (87). Some researchers will argue that to evaluate the effects of interventions it is better to have three groups: an intervention, an active control group of a treatment as usual, and a passive control group that does not receive any treatment. Due to ethical issues (both mindfulness and cognitive stimulation are expected to promote positive effects based on previous studies) and the well-known difficulties to recruit a large number of older persons to research protocols, we decided to opt for two groups with a statistically sound calculated sample.

Furthermore, the fact that when multiple constructs are being measured using common methods and surveys may lead to spurious effects due to the measurement instruments rather than to the constructs being measured is well-known. For instance, false correlations are likely to be produced among the items owing to response styles or social desirability.

Despite these limitations, the present study, which uses qualitative and quantitative approaches, may bring a better understanding of how MBIs may improve the quality of life in objective and subjective perspectives within a mixed-methods approach. Psychometrics parameters and qualitative assessment through focus group and the word free evocation technique may bring a range of meaningful data to find out how mindfulness training may work on older people and if the MBHP program is feasible and efficacious when applied for older population in PC.

## Ethics Statement

The studies involving human participants were reviewed and approved by UNIFESP Research Ethics Committees under the number 2.776.081. The trial was registered on ClinicalTrials.gov under the number NCT03706807 on October 11th 2018. The patients/participants provided their written informed consent to participate in this study.

## Author Contributions

MD, MM, DO, and LR co-conceptualized and designed the study. MM wrote the manuscript draft. SA planned the statistical analysis. VS and VD'A gave support on planning the qualitative assessment and its analysis. LR was the coordinator of the Center for Aging Research where the study will be conducted. AB and LT were leading the control group. MD and LR will supervise the whole study as principal investigators. All authors revised the approved the final version of the manuscript.

## Conflict of Interest

The authors declare that the research was conducted in the absence of any commercial or financial relationships that could be construed as a potential conflict of interest.
