# Supplementary material for: Protocol for a Nested Randomized Controlled Trial to Evaluate the Feasibility and Preliminary Efficacy of the Mindfulness Based Health Promotion Program on the Quality of Life of Older Adults Assisted in Primary Care—“The MBHP-Elderly Study”
Source: Front Med (Lausanne). 2020 Nov 30;7:563099. doi: 10.3389/fmed.2020.563099 (PMC7793825; doi:10.3389/fmed.2020.563099)
Supplement: Supplementary file 1 [file Data_Sheet_1.PDF]

WHOQOL – ABREVIADO

Este questionário é sobre como você se sente a respeito de sua qualidade de vida, saúde e outras áreas de sua vida. Por favor responda a todas as questões. Se você não tem certeza sobre que resposta dar em uma questão, por favor, escolha entre as alternativas a que lhe parece mais apropriada. Esta, muitas vezes, poderá ser sua primeira escolha. Tenha em mente seus valores, aspirações, prazeres e preocupações.
Por favor, leia cada questão, veja o que você acha e circule no número que lhe parece a melhor resposta.

		**muito ruim**	**ruim**	**nem ruim nem boa**	**boa**	**muito boa**
**1**	Como você avaliaria sua qualidade de vida?	1	2	3	4	5
		**muito insatisfeito**	**Insatisfei-to**	**nem satisfeito nem insatisfeito**	**satisfei-to**	**muito satisfeito**
**2**	Quão satisfeito (a) você está com a sua saúde?	1	2	3	4	5

As questões seguintes são sobre **o quanto** você tem sentido algumas coisas nas **últimas duas semanas**.

		**nada**	**muito pouco**	**mais ou menos**	**bastante**	**extrema-mente**
**3**	Em que medida você acha que sua dor (física) impede você de fazer o que você precisa?	1	2	3	4	5
**4**	O quanto você precisa de algum tratamento médico para levar sua vida diária?	1	2	3	4	5
**5**	O quanto você aproveita a vida?	1	2	3	4	5
**6**	Em que medida você acha que a sua vida tem sentido?	1	2	3	4	5
**7**	O quanto você consegue se concentrar?	1	2	3	4	5
**8**	Quão seguro (a) você se sente em sua vida diária?	1	2	3	4	5
**9**	Quão saudável é o seu ambiente físico (clima, barulho, poluição, atrativos)?	1	2	3	4	5

As questões seguintes perguntam sobre **quão completamente** você tem sentido ou é capaz de fazer certas coisas nestas **últimas duas semanas**.

		**nada**	**muito pouco**	**médio**	**muito**	**completa-mente**
**10**	Você tem energia suficiente	1	2	3	4	5

	para seu dia-a- dia?					
**11**	Você é capaz de aceitar sua aparência física?	1	2	3	4	5
**12**	Você tem dinheiro suficiente para satisfazer suas necessidades?	1	2	3	4	5
**13**	Quão disponíveis para você estão as informações que precisa no seu dia-a-dia?	1	2	3	4	5
**14**	Em que medida você tem oportunidades de atividade de lazer?	1	2	3	4	5

As questões seguintes perguntam sobre **quão bem ou satisfeito** você se sentiu a respeito de vários aspectos de sua vida nas **últimas duas semanas**.

		**muito ruim**	**ruim**	**nem ruim nem bom**	**bom**	**muito bom**
**15**	Quão bem você é capaz de se locomover?	1	2	3	4	5
		**muito insatisfei-to**	**insatis-feito**	**nem satisfeito nem insatisfeito**	**satisfei-to**	**muito satisfeito**
**16**	Quão satisfeito (a) você está com o seu sono?	1	2	3	4	5
**17**	Quão satisfeito (a) você está com sua capacidade de desempenhar as atividades do seu dia-a-dia?	1	2	3	4	5
**18**	Quão satisfeito (a) você está com sua capacidade para o trabalho?	1	2	3	4	5
**19**	Quão satisfeito (a) você está consigo mesmo?	1	2	3	4	5
		**muito insatisfei-to**	**insatis-feito**	**nem satisfeito nem insatisfeito**	**satisfei-to**	**muito satisfeito**
**20**	Quão satisfeito (a) você está com as suas relações pessoais (amigos, parentes conhecidos, colegas)?	1	2	3	4	5

**21**	Quão satisfeito (a) você está com sua vida sexual?	1	2	3	4	5
**22**	Quão satisfeito (a) você está com o apoio que você recebe de seus amigos?	1	2	3	4	5
**23**	Quão satisfeito (a) você está com as condições do local onde mora?	1	2	3	4	5
**24**	Quão satisfeito (a) você está com o seu acesso aos serviços de saúde?	1	2	3	4	5
**25**	Quão satisfeito (a) você está com o seu meio de transporte?	1	2	3	4	5

As questões seguintes referem-se à **com que frequência** você sentiu ou experimentou certas coisas nas últimas duas semanas.

		**nunca**	**algumas vezes**	**frequen-temente**	**muito frequen-temente**	**sempre**
**26**	Com que freqüência você tem sentimentos negativos tais como mau humor, desespero, ansiedade, depressão?	1	2	3	4	5

WHOQOL-OLD

Este questionário pergunta a respeito dos seus pensamentos, sentimentos e sobre certos aspectos de sua qualidade de vida, e aborda questões que podem ser importantes para você como membro mais velho da sociedade.
Por favor, tenha em mente os seus valores, esperanças, prazeres e preocupações.
Pedimos que pense na sua vida **nas duas últimas semanas**.

OLD_1. Até que ponto as perdas nos seus sentidos (por exemplo, audição, visão, paladar, olfato, tato), afeta a sua vida diária?

nada	muito pouco	mais ou menos	bastante	extremamente
1	2	3	4	5

OLD_2 . Até que ponto a perda de, por exemplo, audição, visão, paladar, olfato, tato, afeta a sua capacidade de participar em atividades?

nada	muito pouco	mais ou menos	bastante	extremamente
1	2	3	4	5

OLD_3. Quanta liberdade você tem de tomar as suas próprias decisões?

nada	muito pouco	mais ou menos	bastante	extremamente
1	2	3	4	5

OLD_4. Até que ponto você sente que controla o seu futuro?

nada	muito pouco	mais ou menos	bastante	extremamente
1	2	3	4	5

OLD_5. O quanto você sente que as pessoas ao seu redor respeitam a sua liberdade?

nada	muito pouco	mais ou menos	bastante	extremamente
1	2	3	4	5

OLD_6. Quão preocupado você está com a maneira pela qual irá morrer?

nada	muito pouco	mais ou menos	bastante	extremamente
1	2	3	4	5

OLD_7. O quanto você tem medo de não poder controlar a sua morte?

nada	muito pouco	mais ou menos	bastante	extremamente
1	2	3	4	5

OLD_8. O quanto você tem medo de morrer?

nada	muito pouco	mais ou menos	bastante	extremamente
1	2	3	4	5

OLD_9. O quanto você teme sofrer dor antes de morrer?

nada	muito pouco	mais ou menos	bastante	extremamente
1	2	3	4	5

As seguintes questões perguntam sobre **quão completamente** você fez ou se sentiu apto a fazer algumas coisas nas **duas últimas semanas**.

OLD_10. Até que ponto o funcionamento dos seus sentidos (por exemplo, audição, visão, paladar, olfato, tato) afeta a sua capacidade de interagir com outras pessoas?

nada	muito pouco	médio	muito	completamente
1	2	3	4	5

OLD_11. Até que ponto você consegue fazer as coisas que gostaria de fazer?

nada	muito pouco	médio	muito	completamente
1	2	3	4	5

OLD_12. Até que ponto você está satisfeito com as suas oportunidades para continuar alcançando outras realizações na sua vida?

nada	muito pouco	médio	muito	completamente
1	2	3	4	5

OLD_13. O quanto você sente que recebeu o reconhecimento que merece na sua vida?

nada	muito pouco	médio	muito	completamente
1	2	3	4	5

OLD_14. Até que ponto você sente que tem o suficiente para fazer em cada dia?

nada	muito pouco	médio	muito	completamente
1	2	3	4	5

As seguintes questões pedem a você que diga o quanto você se sentiu **satisfeito, feliz ou bem** sobre vários aspectos de sua vida nas **duas últimas semanas**.

OLD_15. Quão satisfeito você está com aquilo que alcançou na sua vida?

muito insatisfeito	insatisfeito	nem satisfeito nem insatisfeito	satisfeito	muito satisfeito
1	2	3	4	5

OLD_16. Quão satisfeito você está com a maneira com a qual você usa o seu tempo?

muito insatisfeito	insatisfeito	nem satisfeito nem insatisfeito	satisfeito	muito satisfeito
1	2	3	4	5

OLD_17. Quão satisfeito você está com o seu nível de atividade?

muito insatisfeito	insatisfeito	nem satisfeito nem insatisfeito	satisfeito	muito satisfeito
1	2	3	4	5

OLD_18. Quão satisfeito você está com as oportunidades que você tem para participar de atividades da comunidade?

muito insatisfeito	insatisfeito	nem satisfeito nem insatisfeito	satisfeito	muito satisfeito
1	2	3	4	5

OLD_19. Quão feliz você está com as coisas que você pode esperar daqui para frente?

muito infeliz	infeliz	nem feliz nem infeliz	feliz	muito feliz
1	2	3	4	5

OLD_20. Como você avaliaria o funcionamento dos seus sentidos (por exemplo, audição, visão, paladar, olfato, tato)?

muito ruim	ruim	nem ruim nem bom	bom	muito bom
1	2	3	4	5

As seguintes questões se referem a qualquer **relacionamento íntimo** que você possa ter.
Por favor, considere estas questões não pensando em relacionamento sexual e sim em relação a um companheiro ou uma pessoa próxima com a qual você pode compartilhar (dividir) sua intimidade (preocupações, sentimentos, etc.) mais do que com qualquer outra pessoa em sua vida. (pode ser, por exemplo um amigo (a), parente.)

OLD_21. Até que ponto você tem um sentimento de companheirismo em sua vida?

nada	muito pouco	mais ou menos	bastante	extremamente
1	2	3	4	5

OLD_22. Até que ponto você sente amor em sua vida?

nada	muito pouco	mais ou menos	bastante	extremamente
1	2	3	4	5

OLD_23. Até que ponto você tem oportunidades para amar?

nada	muito pouco	médio	muito	completamente
1	2	3	4	5

OLD_24. Até que ponto você tem oportunidades para ser amado?

nada	muito pouco	médio	muito	completamente
1	2	3	4	5

Mente Aberta
MINDFULNESS BRASIL

Versão brasileira da MAAS (Mindful Attention Awareness Scale) e instruções para pontuação (Barros et al, 2015)

Há um conjunto de sentenças abaixo sobre a sua experiência diária. Usando a escala de 1-6, por favor, indique a frequência com que você tem cada experiência, atualmente. Por favor, responda de acordo com o que realmente reflita a sua experiência, ao invés de o que você pensa que a sua experiência deveria ser. Por favor, pense em cada item separadamente dos outros.

1	2	3	4	5	6
Quase sempre	Muito frequentemente	Relativamente frequente	Raramente	Muito Raramente	Quase nunca

1	Eu poderia experimentar alguma emoção e só tomar consciência dela algum tempo depois.	1	2	3	4	5	6
2	Eu quebro ou derramo as coisas por falta de cuidado, falta de atenção, ou por estar pensando em outra coisa.	1	2	3	4	5	6
3	Eu tenho dificuldade de permanecer focado no que está acontecendo no presente.	1	2	3	4	5	6
4	Eu costumo andar rápido para chegar ao meu destino, sem prestar atenção ao que eu vivencio no caminho.	1	2	3	4	5	6
5	Eu não costumo notar as sensações de tensão física ou de desconforto até que elas realmente chamem a minha atenção.	1	2	3	4	5	6
6	Eu esqueço o nome das pessoas quase imediatamente após eu tê-lo ouvido pela primeira vez.	1	2	3	4	5	6
7	Parece que eu estou “funcionando no piloto automático”, sem muita consciência do que estou fazendo.	1	2	3	4	5	6
8	Eu realizo as atividades de forma apressada, sem estar realmente atento a elas.	1	2	3	4	5	6
9	Eu fico tão focado no objetivo que quero atingir, que perco a noção do que estou fazendo agora para chegar lá.	1	2	3	4	5	6
10	Eu realizo trabalhos e tarefas automaticamente, sem estar consciente do que estou fazendo.	1	2	3	4	5	6
11	Eu me percebo ouvindo alguém falar e fazendo outra coisa ao mesmo tempo.	1	2	3	4	5	6
12	Eu dirijo no “piloto automático” e depois penso porque eu fui naquela direção.	1	2	3	4	5	6
13	Encontro-me preocupado com futuro ou com o passado.	1	2	3	4	5	6
14	Eu me pego fazendo coisas sem prestar atenção.	1	2	3	4	5	6
15	Eu como sem estar consciente do que estou comendo.	1	2	3	4	5	6

Self-Compassion Scale

Por favor, leia com cuidado antes de responder. Para cada frase, marque o número que mostra com que frequência você se comporta da forma descrita. Use a escala de 1 até 5 para marcar sua escolha, sendo que 1 corresponde a "quase nunca" (QN), e 5 significa "quase sempre" (QS). Não existem respostas certas ou erradas. Gostaríamos de sua opinião pessoal. Você pode escolher qualquer número de 1 até 5.

**Por favor, para cada frase, marque com um "X" a sua resposta.**	**QN**				**QS**
	**1**	**2**	**3**	**4**	**5**
1. Sou realmente crítico e severo com meus próprios erros e defeitos.					
2. Quando fico "pra baixo", não consigo parar de pensar em tudo que está errado comigo.					
3. Quando as coisas vão mal para mim, vejo as dificuldades como parte da vida e que acontecem com todo mundo.					
4. Quando penso nos meus defeitos, eu me sinto realmente isolado do resto do mundo.					
5. Tento ser amável comigo quando me sinto emocionalmente mal.					
6. Quando eu falho em algo importante para mim, fico totalmente consumido por sentimentos de incompetência.					
7. Quando me sinto realmente mal, lembro que há outras pessoas no mundo se sentindo como eu.					
8. Quando as coisas estão realmente difíceis, costumo ser duro comigo mesmo.					
9. Quando algo me deixa aborrecido, tento buscar equilíbrio emocional.					
10. Quando percebo que fui inadequado, tento lembrar que a maioria das pessoas também passa por isso.					
11. Sou intolerante e impaciente com os aspectos de que não gosto na minha personalidade.					
12. Quando estou passando por um momento realmente difícil, eu me dou o apoio e o cuidado de que preciso.					
13. Quando fico "pra baixo", sinto que a maioria das pessoas é mais feliz do que eu.					

14. Quando algo doloroso acontece, tento ver a situação de forma equilibrada.					
15. Tento entender meus defeitos como parte da condição humana.					
16. Quando vejo características que eu não gosto em mim, sou duro comigo mesmo.					
17. Quando eu falho em algo importante para mim, tento ver as coisas por outro ângulo.					
18. Quando passo por dificuldades emocionais, costumo pensar que as coisas são mais fáceis para as outras pessoas.					
19. Sou bondoso comigo quando estou passando por algum sofrimento.					
20. Quando algo me deixa incomodado, sou completamente tomado por sentimentos negativos.					
21. Costumo ser um pouco insensível comigo quando estou sofrendo.					
22. Quando fico “pra baixo”, tento aceitar e entender meus sentimentos.					
23. Sou tolerante com meus próprios erros e defeitos.					
24. Quando algo doloroso acontece comigo, costumo reagir de forma exagerada.					
25. Quando eu falho em algo importante para mim, costumo me sentir muito sozinho nessa situação.					
26. Tento ser compreensivo e paciente com os aspectos da minha personalidade dos quais não gosto.					

DASS – 21 Versão traduzida e validada para o português do Brasil
Autores: Vignola, R.C.B. & Tucci, A.M.

Instruções
Por favor, leia cuidadosamente cada uma das afirmações abaixo e circule o número apropriado **0,1,2 ou 3** que indique o quanto ela se aplicou a você durante a última semana, conforme a indicação a seguir:

0 Não se aplicou de maneira alguma
1 Aplicou-se em algum grau, ou por pouco de tempo
2 Aplicou-se em um grau considerável, ou por uma boa parte do tempo
3 Aplicou-se muito, ou na maioria do tempo

1	Achei difícil me acalmar	0 1 2 3
2	Senti minha boca seca	0 1 2 3
3	Não consegui vivenciar nenhum sentimento positivo	0 1 2 3
4	Tive dificuldade em respirar em alguns momentos (ex. respiração ofegante, falta de ar, sem ter feito nenhum esforço físico)	0 1 2 3
5	Achei difícil ter iniciativa para fazer as coisas	0 1 2 3
6	Tive a tendência de reagir de forma exagerada às situações	0 1 2 3
7	Senti tremores (ex. nas mãos)	0 1 2 3
8	Senti que estava sempre nervoso	0 1 2 3
9	Preocupei-me com situações em que eu pudesse entrar em pânico e parecesse ridículo (a)	0 1 2 3
10	Senti que não tinha nada a desejar	0 1 2 3
11	Senti-me agitado	0 1 2 3
12	Achei difícil relaxar	0 1 2 3
13	Senti-me depressivo (a) e sem ânimo	0 1 2 3
14	Fui intolerante com as coisas que me impediam de continuar o que eu estava fazendo	0 1 2 3
15	Senti que ia entrar em pânico	0 1 2 3
16	Não consegui me entusiasmar com nada	0 1 2 3
17	Senti que não tinha valor como pessoa	0 1 2 3
18	Senti que estava um pouco emotivo/sensível demais	0 1 2 3
19	Sabia que meu coração estava alterado mesmo não tendo feito nenhum esforço físico (ex. aumento da frequência cardíaca, disritmia cardíaca)	0 1 2 3
20	Senti medo sem motivo	0 1 2 3
21	Senti que a vida não tinha sentido	0 1 2 3

Escala de Religiosidade da Universidade Duke (DUREL)

1. Com que frequência você vai a uma igreja, templo ou outro encontro religioso?

1. Mais do que uma vez por semana
2. Uma vez por semana
3. Duas a três vezes por mês
4. Algumas vezes por ano
5. Uma vez por ano ou menos
6. Nunca

2. Com que frequência você dedica o seu tempo a atividades religiosas individuais, como preces, rezas, meditações, leitura da bíblia ou de outros textos religiosos?

1. Mais do que uma vez ao dia
2. Diariamente
3. Duas ou mais vezes por semana
4. Uma vez por semana
5. Poucas vezes por mês
6. Raramente ou nunca

A seção seguinte contém 3 frases a respeito de crenças ou experiências religiosas. Por favor, anote o quanto cada frase se aplica a você.

3. Em minha vida, eu sinto a presença de Deus (ou do Espírito Santo).

1. Totalmente verdade para mim
2. Em geral é verdade
3. Não estou certo
4. Em geral não é verdade
5. Não é verdade

4. As minhas crenças religiosas estão realmente por trás de toda a minha maneira de viver.

1. Totalmente verdade para mim
2. Em geral é verdade
3. Não estou certo
4. Em geral não é verdade
5. Não é verdade

5. Eu me esforço muito para viver a minha religião em todos os aspectos da vida.

1. Totalmente verdade para mim
2. Em geral é verdade
3. Não estou certo
4. Em geral não é verdade
5. Não é verdade

INDÍCE DE QUALIDADE DO SONO DE PITTSBURGH - PSQI
PITTSBURGH SLEEP QUALITY INDEX

Nome:____ Coleta:___/____/_______
Idade:_____________ Sexo:____

Instruções:
As seguintes perguntas são relativas aos seus hábitos usuais de sono durante o **último mês somente**. Suas respostas devem indicar a lembrança mais exata da **maioria** dos dias e noites no último mês. Por favor, responda a todas as perguntas.

1. Durante o mês passado, a que horas você foi deitar à noite, na maioria das vezes?
   Hora usual de deitar________________

2. Durante o mês passado, quanto tempo (em minutos) você demorou para pegar no sono na maioria das vezes?
   Número de minutos ________________

3. Durante o mês passado, a que horas você geralmente levantou de manhã.
   Hora usual de levantas_________________

4. Durante o mês passado, quantas horas de sono por noite você dormiu? (pode ser diferente do número de horas que você ficou na cama)
   Horas de sono por noite ________________

Para cada uma das questões seguintes, escolha uma única resposta, que você ache mais correta. Por favor, responda todas as questões.

5. Durante o mês passado, com que frequência você **teve dificuldade de dormir** porque você...
   a) Não conseguiu adormecer em até 30 minutos
      ( ) Nenhuma no último mês
      ( ) Menos de 1 vez por semana
      ( ) 1 ou 2 vezes por semana
      ( ) 3 ou mais vezes por semana

   b) Acordou no meio da noite ou muito cedo pela manhã
      ( ) Nenhuma no último mês
      ( ) Menos de 1 vez por semana
      ( ) 1 ou 2 vezes por semana
      ( ) 3 ou mais vezes por semana

   c) Precisou levantar para ir ao banheiro
      ( ) Nenhuma no último mês
      ( ) Menos de 1 vez por semana
      ( ) 1 ou 2 vezes por semana
      ( ) 3 ou mais vezes por semana

   d) Teve dificuldade para respirar
      ( ) Nenhuma no último mês
      ( ) Menos de 1 vez por semana
      ( ) 1 ou 2 vezes por semana
      ( ) 3 ou mais vezes por semana

   e) Tossiu ou roncou alto
      ( ) Nenhuma no último mês
      ( ) Menos de 1 vez por semana
      ( ) 1 ou 2 vezes por semana
      ( ) 3 ou mais vezes por semana

f) Sentiu muito frio
( ) Nenhuma no último mês
( ) Menos de 1 vez por semana
( ) 1 ou 2 vezes por semana
( ) 3 ou mais vezes por semana

g) Sentiu muito calor
( ) Nenhuma no último mês
( ) Menos de 1 vez por semana
( ) 1 ou 2 vezes por semana
( ) 3 ou mais vezes por semana

h) Teve sonhos ruins ou pesadelos
( ) Nenhuma no último mês
( ) Menos de 1 vez por semana
( ) 1 ou 2 vezes por semana
( ) 3 ou mais vezes por semana

i) Sentiu dores
( ) Nenhuma no último mês
( ) Menos de 1 vez por semana
( ) 1 ou 2 vezes por semana
( ) 3 ou mais vezes por semana

j) Outra(s) razão(ões), por favor descreva:____
____

Com que frequência você teve dificuldade para dormir devido a esta razão:
( ) Nenhuma no último mês
( ) Menos de 1 vez por semana
( ) 1 ou 2 vezes por semana
( ) 3 ou mais vezes por semana

6. Durante o mês passado como você classificaria a qualidade do seu sono de uma maneira geral?
( ) Muito boa
( ) Boa
( ) Ruim
( ) Muito ruim

7. Durante o mês passado com que frequência você tomou medicamento (prescrito ou "por conta própria") para lhe ajudar a dormir?
( ) Nenhuma no último mês
( ) Menos de 1 vez por semana
( ) 1 ou 2 vezes por semana
( ) 3 ou mais vezes por semana

8. Durante o mês passado, com que frequência você teve problemas para ficar acordado enquanto dirigia, comia ou participava de uma atividade social (festa, reunião de amigos, trabalho ou estudo)
( ) Nenhuma no último mês
( ) Menos de 1 vez por semana
( ) 1 ou 2 vezes por semana
( ) 3 ou mais vezes por semana

9. Durante o mês passado, você sentiu indisposição ou falta de ânimo para realizar suas atividades diárias?
( ) Nenhuma indisposição nem falta de ânimo
( ) Pequena indisposição e falta de ânimo
( ) Moderada indisposição e falta de ânimo
( ) Muita indisposição e falta de ânimo

10. Você tem um(a) parceiro(a) ou colega de quarto?
( ) Não
( ) Parceiro ou colega, mas em outro quarto
( ) Parceiro no mesmo quarto, mas não na mesma cama
( ) Parceiro na mesma cama

Se você tem um(a) parceiro(a) ou colega de quarto, pergunte a ele(a) com que frequência no último mês você teve...

a) Ronco alto:
( ) Nenhuma no último mês
( ) Menos de 1 vez por semana
( ) 1 ou 2 vezes por semana
( ) 3 ou mais vezes por semana

b) Longas paradas na respiração enquanto dormia:
( ) Nenhuma no último mês
( ) Menos de 1 vez por semana
( ) 1 ou 2 vezes por semana
( ) 3 ou mais vezes por semana

c) Contrações ou puxões nas pernas enquanto você dormia:
( ) Nenhuma no último mês
( ) Menos de 1 vez por semana
( ) 1 ou 2 vezes por semana
( ) 3 ou mais vezes por semana

d) Episódios de desorientação ou de confusão durante o sono:
( ) Nenhuma no último mês
( ) Menos de 1 vez por semana
( ) 1 ou 2 vezes por semana
( ) 3 ou mais vezes por semana

e) Outras alterações (inquietações) enquanto você dorme; por favor, descreva:_________
____
( ) Nenhuma no último mês
( ) Menos de 1 vez por semana
( ) 1 ou 2 vezes por semana
( ) 3 ou mais vezes por semana

MONTREAL COGNITIVE ASSESSMENT (MOCA)
Versão Experimental Brasileira

Nome:______________________ Data de nascimento: __/__/__
Escolaridade:__________________ Data de avaliação: __/__/__
Sexo:______________________ Idade:_____

Seção	Conteúdo	Pontos
**VISUOESPACIAL / EXECUTIVA**	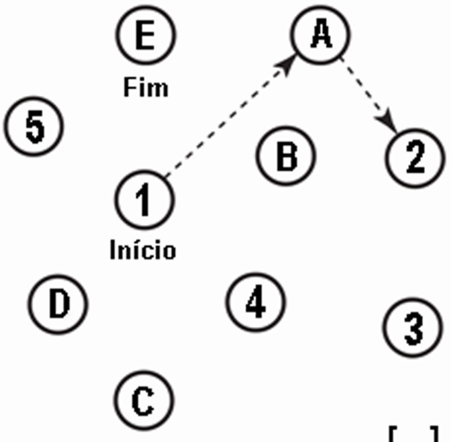 [ ] · 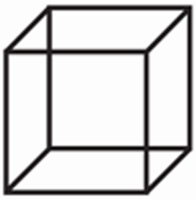 Copiar o cubo [ ] · Desenhar um RELÓGIO (onze horas e dez minutos) (3 pontos) [ ] Contorno [ ] Números [ ] Ponteiros	__/5
**NOMEAÇÃO**	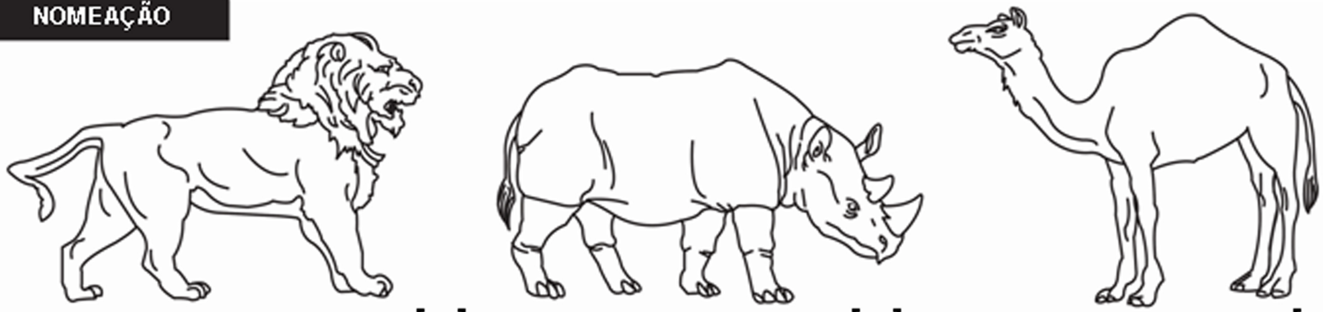 [ ] [ ] [ ]	__/3

**MEMÓRIA**		Rosto	Veludo	Igreja	Margarida	Vermelho	Sem Pontuação
Leia a lista de palavras, O sujeito de repeti-la, faça duas tentativas Evocar após 5 minutos	1ª tentativa						
	2ª tentativa						

Seção	Conteúdo	Pontos
**ATENÇÃO**	Leia a seqüência de números (1 número por segundo) — O sujeito deve repetir a seqüência em ordem direta [ ] 2 1 8 5 4; O sujeito deve repetir a seqüência em ordem indireta [ ] 7 4 2	__/2
	Leia a série de letras. O sujeito deve bater com a mão (na mesa) cada vez que ouvir a letra "A". Não se atribuem pontos se ≥ 2 erros. [ ] F B A C M N A A J K L B A F A K D E A A A J A M O F A A B	__/1
	Subtração de 7 começando pelo 100 [ ] 93 [ ] 86 [ ] 79 [ ] 72 [ ] 65 — 4 ou 5 subtrações corretas: 3 pontos; 2 ou 3 corretas 2 pontos; 1 correta 1 ponto; 0 correta 0 ponto	__/3
**LINGUAGEM**	Repetir: Eu somente sei que é João quem será ajudado hoje. [ ] O gato sempre se esconde embaixo do Sofá quando o cachorro está na sala. [ ]	__/2
	Fluência verbal: dizer o maior número possível de palavras que comecem pela letra F (1 minuto). [ ]______ (N ≥ 11 palavras)	__/1
**ABSTRAÇÃO**	Semelhança p. ex. entre banana e laranja = fruta [ ] trem - bicicleta [ ] relógio - régua	__/2

**EVOCAÇÃO TARDIA**		Rosto	Veludo	Igreja	Margarida	Vermelho		Pontos
	Deve recordar as palavras SEM PISTAS	[ ]	[ ]	[ ]	[ ]	[ ]	Pontuação apenas para evocação SEM PISTAS	__/5
OPCIONAL	Pista de categoria							
	Pista de múltipla escolha							

Seção	Conteúdo	Pontos
**ORIENTAÇÃO**	[ ] Dia do mês [ ] Mês [ ] Ano [ ] Dia da semana [ ] Lugar [ ] Cidade	__/6

TOTAL __/30
Adicionar 1 pt se ≤ 12 anos de escolaridade

© Z. Nasreddine MD www.mocatest.org
Versão experimental Brasileira: *Ana Luisa Rosas Sarmento*
Paulo Henrique Ferreira Bertolucci - José Roberto Wajman
(UNIFESP-SP 2007)
